# Hyperspectral images of grapevine leaves including healthy leaves and leaves with biotic and abiotic symptoms

**DOI:** 10.1038/s41597-023-02642-w

**Published:** 2023-10-26

**Authors:** Maxime Ryckewaert, Daphné Héran, Jean-Philippe Trani, Silvia Mas-Garcia, Carole Feilhes, Fanny Prezman, Eric Serrano, Ryad Bendoula

**Affiliations:** 1 ITAP, Univ Montpellier, INRAE, Institut Agro, Montpellier, France; 2 Inria, LIRMM, University of Montpellier, CNRS, Montpellier, France; 3 IFV, 1920 Route de Lisle-sur-Tarn, 81310 Peyrole, France

**Keywords:** Pattern recognition receptors in plants, Plant sciences, Plant breeding, Image processing

## Abstract

A hyperspectral imaging database was collected on two hundred and five grape plant leaves. Leaves were measured with a hyperspectral camera in the visible/near infrared spectral range under controlled conditions. This dataset contains hyperspectral acquisition of grape leaves of seven different varieties. For each variety, acquisitions were performed on healthy leaves and leaves with foliar symptoms caused by different grapevine diseases showing clear symptoms of biotic or abiotic stress on other organs. For each leaf, chemical measurements such as chlorophyll and flavonol contents were also performed.

## Background & Summary

In a context of population increase, reduction of arable lands and global climatic change, agricultural production still needs to be increased and secured in a durable way with respect to the environment. Improving plant production for food and feed is one of the main challenges for the years to come. To meet this challenge, one of the ways is the early and precise identification of biotic and abiotic symptoms on plant leaves (water stress, disease, or bacteria).

Optical instruments and especially multispectral (MSI) and hyperspectral imaging (HSI) are relevant tools for the automated and non-invasive detection of biotic and abiotic symptoms^1–5^.

HSI is highly informative because it has both high spectral and spatial resolutions leading to an increased use in agricultural applications in recent years. For example, new case studies have emerged in the field of plant breeding^6^, crop monitoring such as water stress detection^7^, maturity monitoring^8^, disease detection^9,10^ or prediction of biochemical traits^11,12^.

Although these various studies are promising, the use of HSI forces current analysis methods to be rethought to exploit this rich amount of information^6,13,14^. The training dataset should be as diverse as possible to enable the learning algorithm to be robust. This is particularly true with biological samples which are complex media containing a high variability of response. The data we proposed is designed to be this new kind of HSI library for an application in agriculture and more specifically for identification and detection of different stress symptoms at the grapevine leaf level. For this purpose, a spectral imaging database^15^ was collected from two hundred and four grapevine leaves. The leaves were measured with a hyperspectral camera in the visible/near infrared spectral range under controlled conditions. For each leaf, foliar contents (chlorophyll, epidermal flavonol and nitrogen) were measured. This dataset contains the reflectance spectra of grape leaves of seven different varieties. For each variety, acquisitions were performed on healthy leaves and leaves with foliar symptoms caused by different grapevine diseases showing clear symptoms of biotic or abiotic stress.

A subset of this database has already been used to produce a new method of HSI data processing. We have recently published this new approach^9^. In this work, combination of multivariate curve resolution-alternating least squares (MCR-ALS) and factorial discriminant analysis (FDA) is proposed to detect the Flavescence dorée grapevine disease from hyperspectral imaging. By making this data available to other researchers, we hope to encourage them to do similar work and proposed new algorithms.

## Methods

### Samples and analyses

Leaves were collected during September 2020, in the south of France (GPS coordinates: 43.84208931745156, 1.8538190583140841). Infected leaves were chosen in order to represent at best the variability of the available symptoms in terms of severity and stage of infection. A similar proportion of the number of leaves of both red and white varieties was collected for this experiment. In total two hundred and four leaves were collected in the fields. All information about leaves and their respective symptoms is summarized in Tables 1–3.

**Table 1:** Number of healthy and infected leaves per variety.

Variety	Number of infected leaves	Number of healthy leaves
Colombard	18	5
Duras	19	3
Fer	20	5
Gamay	39	11
Loin de l’œil	20	5
Mauzac	20	5
Chardonnay	24	11

**Table 2:** Number of images per symptom.

Symptom	Number of leaves
Healthy	40
Flavescence dorée (*Scaphoideus titanus*)	80
Water stress	13
Wood diseases	17
Buffalo treehopper (*Stictocephala bisonia*)	17
Green leafhopper (*Empoasca vitis*)	20
Senescence	8
Deficiency	1
Chlorosis	1
Discoloration	1
Damage (hail, harvester)	5
Downy mildew (*Plasmopara viticola*)	2

**Table 3:** Number of images per symptom per variety.

Variety	Symptom	Number of leaves
Gamay	Flavescence dorée	10
Gamay	Healthy	5
Gamay	Water stress	2
Gamay	Buffalo treehopper	2
Gamay	Green leafhopper	3
Gamay	Wood diseases	1
Gamay	Buffalo treehopper	1
Gamay	Green leafhopper	1
Fer	Flavescence dorée	10
Fer	Healthy	5
Fer	Water stress	5
Fer	Buffalo treehopper	3
Fer	Wood diseases	1
Fer	Green leafhopper	1
Duras	Flavescence dorée	9
Duras	Healthy	3
Duras	Water stress	2
Duras	Buffalo treehopper	3
Duras	Green leafhopper	3
Duras	Deficiency	1
Duras	Senescence	1
Gamay	Flavescence dorée	9
Gamay	Healthy	6
Gamay	Water stress	4
Gamay	Buffalo treehopper	5
Gamay	Green leafhopper	1
Colombard	Flavescence dorée	10
Colombard	Healthy	5
Colombard	Wood diseases	4
Colombard	Green leafhopper	2
Colombard	Chlorosis	1
Colombard	Discoloration	1
Loin de l’œil	Flavescence dorée	10
Mauzac	Flavescence dorée	10
Loin de l’œil	Damaged	5
Loin de l’œil	Wood diseases	3
Loin de l’œil	Green leafhopper	4
Loin de l’œil	Senescence	3
Mauzac	Healthy	5
Mauzac	Buffalo treehopper	3
Mauzac	Mildew	2
Mauzac	Wood diseases	2
Mauzac	Green leafhopper	1
Mauzac	Senescence	2
Chardonnay	Flavescence dorée	12
Chardonnay	Healthy	11
Chardonnay	Wood diseases	6
Chardonnay	Senescence	1
Chardonnay	Green leafhopper	4

Each leaf and each vine from which it was extracted were diagnosed by a phytopathology expert. Leaves were extracted from the front face, in the middle of the canopy to avoid the younger and older organs which can present a different physiological behaviour. Regarding healthy leaves, they were selected in the same regions and they were asserted absent of any symptom. However, some of the healthy sample can exhibit slight forms of mechanical or chemical wounds (due to protection, management operations) and some slight damage caused by insects. In order to guarantee that leaf physiological status were not affected by the time delay between collection and acquisition, leaves were carried in controlled temperature and hydric conditions.

### Foliar content measurements

**Fig. 1:**
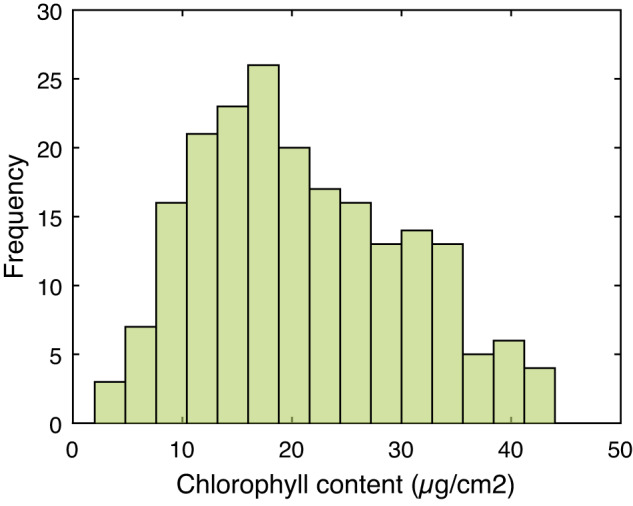
Histogram of chlorophyll content values.

**Fig. 2:**
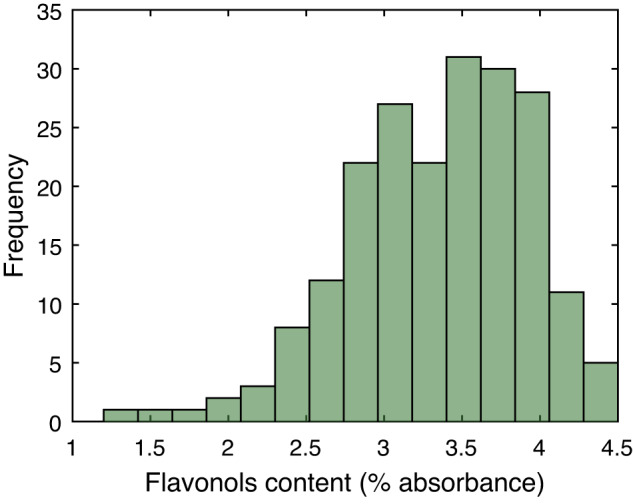
Histogram of flavonols content values.

**Fig. 3:**
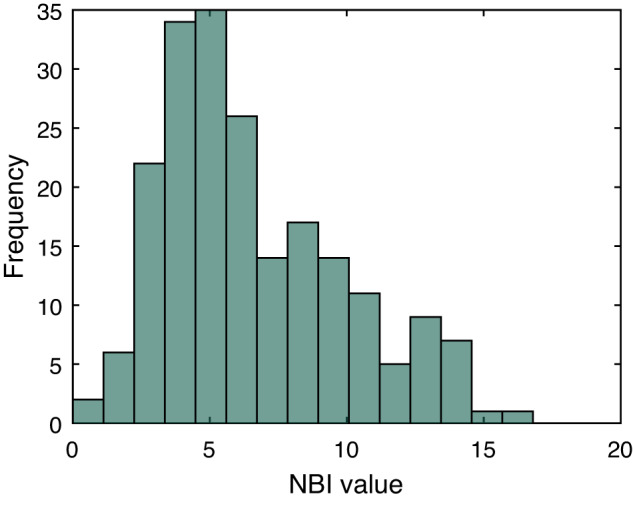
Histogram of NBI values.

For each leaf, foliar content measurements (see Figs. 1–3) were made before sampling. These measurements were carried out with a Dualex 311 scientific+ TM (Force-A, Orsay, France) to provide chlorophyll a + b content (µg/cm2), epidermal flavonols content (in % of relative absorbance) and the crop nitrogen status index (NBI).

### Hyperspectral image acquisition

Hyperspectral images were acquired on each individual leaf under controlled conditions in laboratory. Acquisitions of leaf images were performed with a hyperspectral camera (IQ, Specim, Finland). Imaging of grapevine leaves was carried out in the spectral range of 400–900 nm, with a spectral resolution of 7 nm. Illumination was provided by a halogen lamp (Arrilite 750 Plus ARRI, Munich, Germany) and constant angles of −50° and 50° were maintained between the axes of the halogen lamp and the axis of the hyperspectral camera.

For each sample image, the intensity of the reflected light *I*(*λ*) was measured. The dark current *I*_*d*_(*λ*) i.e. signal without light, was recorded for each acquisition and then subtracted. The intensity *I*_0_(*λ*) of the light reflected by a certified standard reference (Labsphere, SRS-40-010) was measured to standardise spectra and to prevent from non-linearities of all the instrumentation components (light source, lens, fibers and spectrometer). From these measurements, a reflectance image *R*(*λ*) was calculated for each sample, as follows:

1 $$R(\lambda )=\frac{I(\lambda )-{I}_{d}(\lambda )}{{I}_{0}(\lambda )-{I}_{d}(\lambda )}$$

Where *λ* is the wavelength, *I*(*λ*) and *I*_0_(*λ*) are the images of the reflected light intensity from the sample and from the reference respectively, and *I*_*d*_(*λ*) is the image in dark

## Data Records

The dataset, mentioned scripts and algorithms are available in the INRAE data repository^15^.

This dataset contains two table files (description.csv and description_variables.csv), a folder called *Data/* and another folder called *Code/*. The first table file (description.csv) contains the experiment factors and the reference measurements. The second table file (description_variables.csv) contains information about variables used in the first table file.

**Fig. 4:**
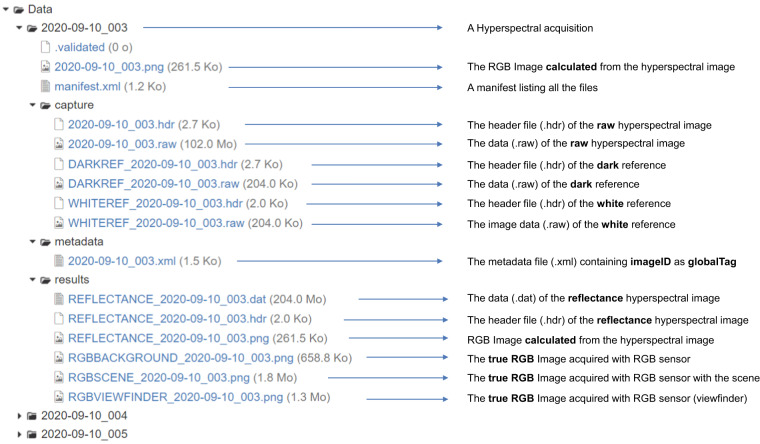
Tree structure of acquisition files.

**Fig. 5:**
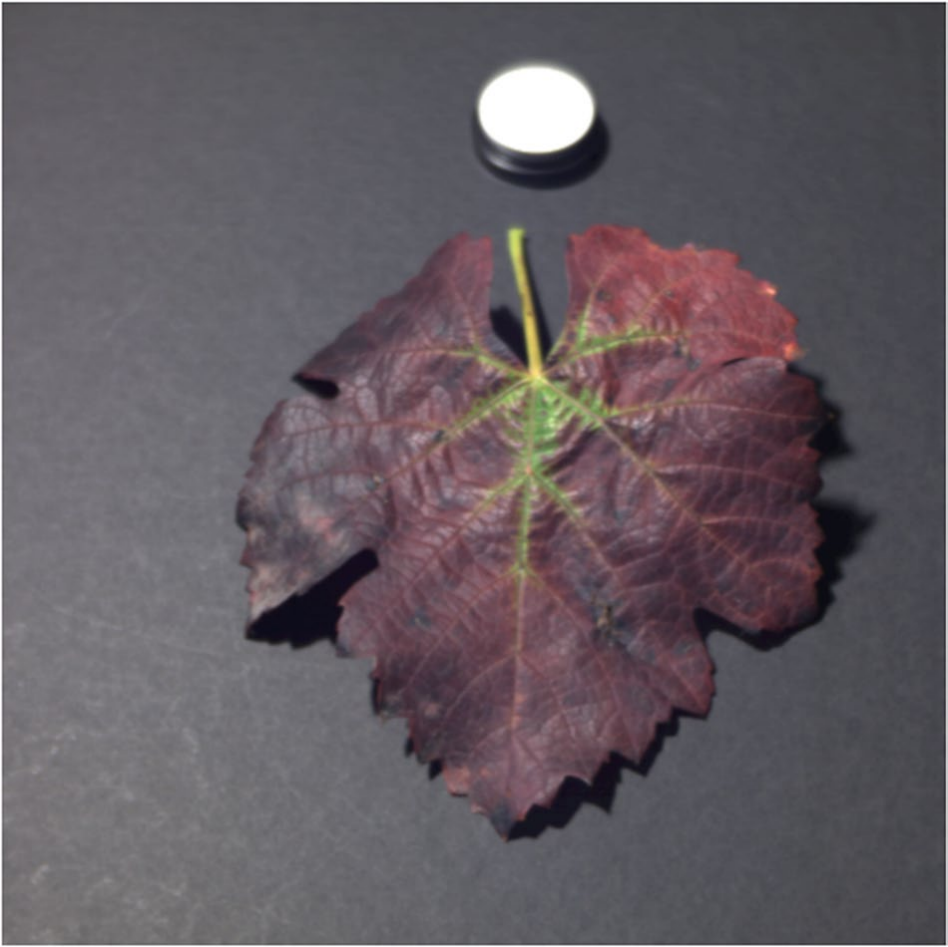
An RGB image reconstructed from a hyperspectral image.

The folder ‘*Data/*’ contains 204 folders corresponding to 204 hyperspectral image acquisitions. In this data directory (see Fig. 4), each folder is named with the acquisition date followed by the acquisition number (YYYY-MM-DD_NBR). Reflectance image files are located in the ‘results’ directories and the acquisition date and acquisition number are specified in the file name (REFLECTANCE_YYYY-MM-DD_NBR.dat). These reflectance images are stored in ENVI format containing binary data (.dat) and header file (.hdr). Each reflectance image (.dat) is around 214 MB. The first and the second dimensions correspond to a spatial position (pixels) forming the image composed of 512 × 512 pixels (see Fig. 5). The third dimension refers to spectral variables with two hundred and four spectral bands.

For each image acquisition, raw image, white reference and dark measurements are available in the directory named ‘capture’. All these raw data are also stored in ENVI format.

For each hyperspectral acquisition, a metadata file is produced containing information about the acquisition. In this metadata file, an identification key called **global_tag** allows the image to be linked to factors in the experimentation or to reference values.

## Technical Validation

We analysed part of this dataset in a first publication to classify images of diseased (flavescence dorée) and healthy leaves^9^. In this study, we proposed a methodology based on multivariate curve resolution-alternating least squares (MCR-ALS) and factorial discriminant analysis (FDA). In this publication we tried to classify each leaf pixel for each image. For the total pixels to be classified (both infected and healthy pixels), the classification rate achieved 85.1%. Another classification result at the leaf (image) level was also investigated. The classification of an image was obtained by counting the majority class among the image pixels. Out of the thirty seven test images, only two images were misclassified with this method.

**Fig. 6:**
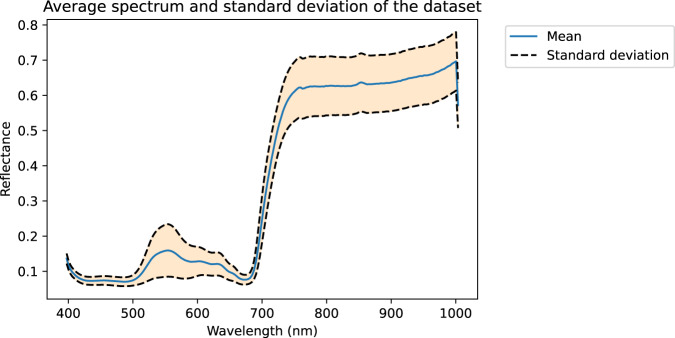
Mean spectrum and standard deviation of all leaf pixels.

**Fig. 7:**
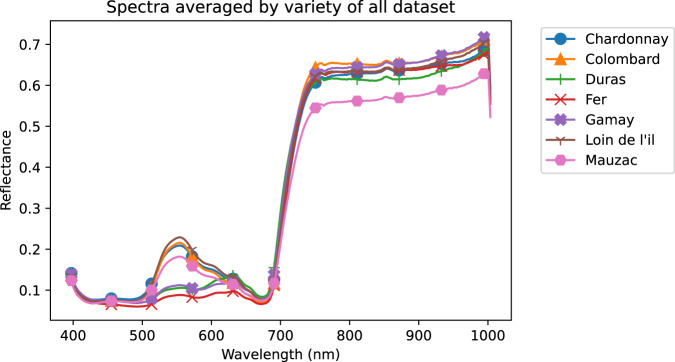
Average spectra per variety.

**Fig. 8:**
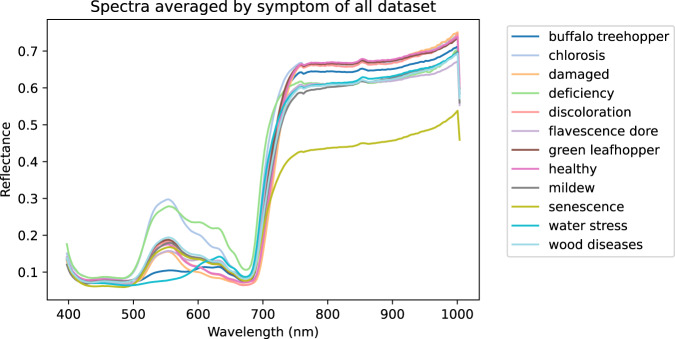
Average spectra per symptom.

The aforementioned publication was based on a subset of the whole dataset. Indeed, the exploitation of this quantity of spectral images to differentiate as many symptoms is a real challenge. Due to the complexity of this dataset and the difficulty of providing masks for each symptom, for initial exploration the average spectrum of the database (see Fig. 6) is calculated, as well as the average spectra per variety (see Fig. 7) and per symptom (see Fig. 8). Then an exploration of the average spectra per leaf is performed by a Principal Component Analysis (PCA).

### Mean spectrum and standard deviation of the entire database

The mean spectrum and standard deviation are calculated from all spectra of the two hundred and four measured leaves (see Fig. 6). The average spectrum is typical of a vegetation spectrum. Low values between 400 nm and 500 nm are mainly related to carotenoid and chlorophyll (a + b) contents. The characteristic large peak around 550 nm is attributed to the anthocyanin content. The spectral region between 620 nm and 680 nm is related to the chlorophyll content of the leaves. The red edge between 680 and 750 nm is also typical of vegetation, separates the visible spectral region related to pigments and the plateau between 750 and 1000 nm related to the leaf structure.

### Average spectra per variety and per symptom

From this database, average spectra are calculated per variety (see Fig. 7) and per symptom (see Fig. 8). Out of the seven average spectra per variety (see Fig. 7), two spectrum shapes are identified in the spectral region between 500 nm to 700 nm. The three spectra corresponding to ‘Duras’, ‘Fer’ and ‘Gamay’ varieties have lower values around 550 nm while the spectra corresponding to ‘Chardonnay’, ‘Colombard’, ‘Loin de l’œil’ and ‘Mauzac’ have higher values. This difference seems to be related to the anthocyanin content in leaves depending on whether the variety is red or white.

Figure 8 displays the average spectrum for each of the twelve symptoms. The spectrum corresponding to the healthy leaf modality shows the same similarities of a typical vegetation spectrum as described above (see Fig. 6). Although for each symptom the spectra are averaged across all grape varieties, differences are noticeable. For example, ‘deficiency’ and ‘chlorosis’ symptoms differ from other symptoms with higher values from 500 nm to 650 nm. Two other symptoms (‘buffalo treehopper’ and ‘water stress’) also differ in the same spectral range but with lower values. The differences between the average spectra between 600 nm and 700 nm, as well as the dynamics of the red-edge or the shape of the plateau would require further processing.

### Principal component analysis

**Fig. 9:**
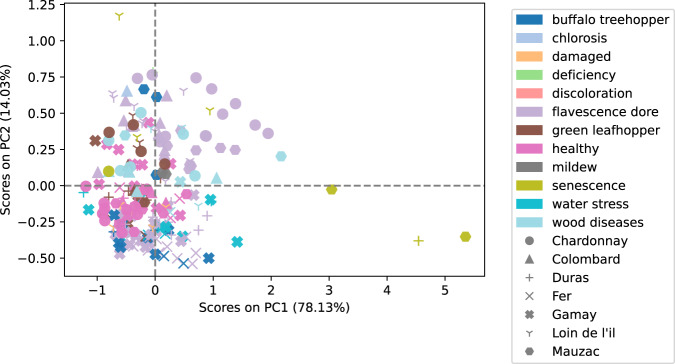
Scores obtained on the two first components.

For each image, an average spectrum was calculated from the leaf pixels. Then a principal component analysis was performed on these two hundred and four average spectra. Figure 9 shows PCA scores obtained for the two first components. A few combinations (variety, symptoms) show a particular behaviour on this score plot. For example, scores of ‘Chardonnay’ combined with ‘flavescence dorée’ have positive scores on both axes and are opposite to the negative scores of healthy ‘Chardonnay’ modality. For other varieties and symptoms, scores are more evenly distributed along the two axes. This is can be explained by the preponderance of the ‘flavescence dorée’ and ‘heatlhy’ observations. Another notable observation is the clear distinction of some observations from the rest of the group, such as ‘senescence’ combined with the ‘Duras’ and ‘Mauzac’ variety on PC1 and ‘senescence’ combined with ‘Loin de l’œil’ variety on PC2.

**Fig. 10:**
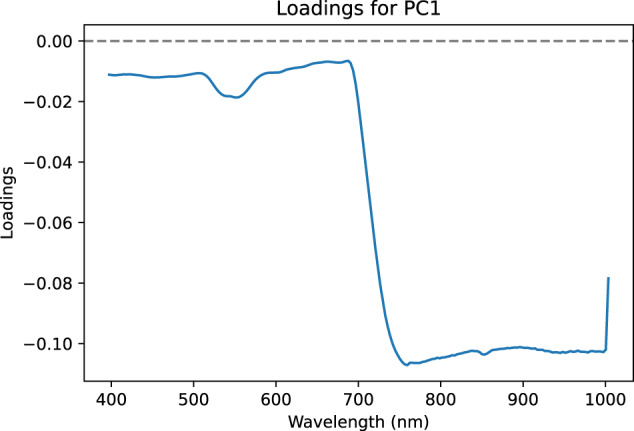
Loadings of the first component.

**Fig. 11:**
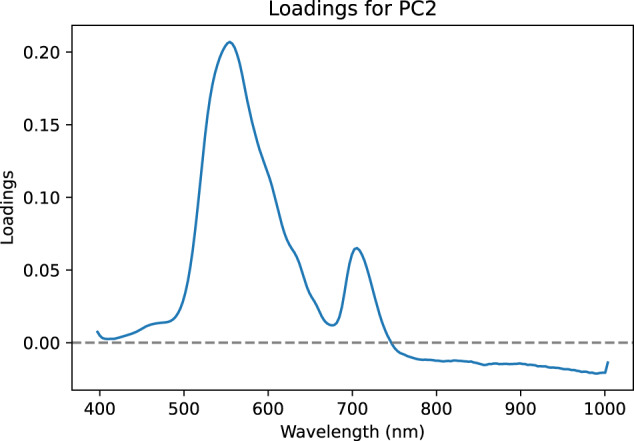
Loadings of the second component.

These results should be considered in relation to the loadings of the principal components concerned (see Figs. 10, 11). The first component corresponds to an inverted overall shape of the spectra (see Fig. 6) which could correspond to the total amount of signal received by the camera. The second component shows loadings of positive values between 400 nm and 600 nm with a strong positive value in the 550 nm region which is related to anthocyanin content. This technical validation was only carried out on the first two principal components of a PCA. The availability of this dataset would allow further study through other principal components or even more generally using other methods. This dataset offers great perspectives for further study, such as classification capabilities according to confounding factors, assessment of spectral variability of symptoms according to variety or improvement of the labelling process by selecting only symptomatic areas of the leaf.

## Usage Notes

There are many advantages to this database. Firstly, it provides hyperspectral images covering a multitude of grapevine symptoms, including different grape varieties. One of the benefits of this dataset lies in the possibility of developing new analysis methods. On a more practical level, it will be used to study the potential of hyperspectral imaging to detect the symptoms proposed and to identify confounding factors. In addition, measurements are carried out under controlled conditions, guaranteeing the reliability and accuracy of the data collected. In particular, measurements are carried out on whole leaves, which generates an abundance of pixels available.

This dataset has certain limitations. Firstly, there may be an imbalance between the number of images available for each grapevine symptom, which could potentially bias the results. Another limitation is that the small number of images available can be limiting for deep learning approaches.

## Data Availability

A Python code example is provided in the *‘Code’* folder. This example helps the reader to understand how to open hyperspectral images, to extract spectra, or to display the results obtained after a Principal Component Analysis (PCA) applied on the first image and a PCA on all average spectra where one average spectrum is obtained per leaf/image.
